# Supplementary material for: Inequality and inequity in network-based ranking and recommendation algorithms
Source: Sci Rep. 2022 Feb 7;12:2012. doi: 10.1038/s41598-022-05434-1 (PMC8821643; doi:10.1038/s41598-022-05434-1)
Supplement: Supplementary file 1 — Supplementary Information. [file 41598_2022_5434_MOESM1_ESM.pdf]

Inequality and Inequity in Network-based Ranking and Recommendation Algorithms

Lisette Espín-Noboa,[1, 2, 3] Claudia Wagner,[4, 5, 1] Markus Strohmaier,[6, 4, 1] and Fariba Karimi[1]
[1] Complexity Science Hub, Vienna, Austria
[2] University of Koblenz-Landau, Koblenz, Germany
[3] Central European University, Vienna, Austria
[4] GESIS – Leibniz-Institute for the Social Sciences, Cologne, Germany
[5] RWTH Aachen University, Aachen, Germany
[6] University of Mannheim, Mannheim, Germany

Supplementary Material

Appendix A: Analytics

1. Derivation of the probability of having an internal link

Let $K_a^{in}(t)$ and $K_a^{out}(t)$ be the sum of the in- and out-degrees of nodes from group a at time t. The overall growth of the network follows a DPAH process. Thus, the evolution of in-degree and out-degree follows:

$$\begin{cases} K_a^{in}(t) + K_b^{in}(t) = K^{in}(t) = mt \\ K_a^{out}(t) + K_b^{out}(t) = K^{out}(t) = mt \end{cases} \tag{A1}$$

where m is the number of new links in the network at each time step t. In each time step, a node v_i is chosen. That results in m new out-going links from v_i. We set $m = 1$. Thus, in each time step only one edge is created from v_i to v_j.

Let us denote the relative fraction of group size for each group as f_a and f_b and their respective activity parameters γ_a and γ_b that represent the exponents of the activity distribution. Thus, the behavior of the network is similar to what we have shown before [1]; only the total number of links is different. Let us also define $\hat{\gamma_a}$ as an average value drawn from the activity distribution of group a, $\rho(\gamma_a) = X^{-\gamma_a}$ using mean field approximation. Similarly, $\hat{\gamma_b}$ as an average value drawn from the activity distribution of group b, $\rho(\gamma_b) = X^{-\gamma_b}$. We can show that in the limit of $\Delta t \to 0$, for each group, the in-degree growth function follows:

$$\frac{dK_a^{in}}{dt} = m\left(f_a \times \hat{\gamma_a}\left(1 + \frac{h_{aa}K_a^{in}(t)}{h_{aa}K_a^{in}(t) + h_{ab}K_b^{in}(t)}\right) + f_b\hat{\gamma_b}\left(\frac{h_{ba}K_a^{in}(t)}{h_{bb}K_b^{in}(t) + h_{ba}K_a^{in}(t)}\right)\right) \tag{A2}$$

$$\frac{dK_b^{in}}{dt} = m\left(f_b \times \hat{\gamma_b}\left(1 + \frac{h_{bb}K_b^{in}(t)}{h_{bb}K_b^{in}(t) + h_{ba}K_a^{in}(t)}\right) + f_a\hat{\gamma_a}\left(\frac{h_{ab}K_b^{in}(t)}{h_{aa}K_a^{in}(t) + h_{ab}K_b^{in}(t)}\right)\right) \tag{A3}$$

Next, we focus on the case of links within group a. The same analysis applies for group b.

Let p_{aa} be the probability to establish a link between two nodes of group a. The probability for an incoming or existing node from group a to link to a node of the same group is given by:

$$p_{aa}(t) = f_a \frac{h_{aa}K_a^{in}(t)}{h_{aa}K_a^{in}(t) + h_{ab}K_b^{in}(t)} \tag{A4}$$

In the simple network growth model, the total degree of the groups increases linearly over time.

$$\begin{cases} K_a^{in}(t) = Cm\hat{\gamma_a}\hat{\gamma_b}t \\ K_b^{in}(t) = (2-C)m\hat{\gamma_a}\hat{\gamma_b}t \\ K_a^{out}(t) = m\hat{\gamma_a}t \\ K_b^{out}(t) = m\hat{\gamma_b}t \end{cases} \tag{A5}$$

Denoting C as the in-degree growth factor of the minority group.

2. Calculating homophily from empirical network

We can calculate homophily in empirical networks using the information about in-group links. First, the total number of edges in a directed network follows:

$$e = e_{aa} + e_{ab} + e_{ba} + e_{bb} \tag{A6}$$

To calculate e_{aa}, the number of links within class a, we can simply argue that it depends on p_{aa}, the probability of connecting two nodes belonging to class a, multiplied by the probability of the arrival or source node to be of class a, denoted by f_a, the fraction of nodes in class a, as shown in Equation (A4).

Our network model grows linearly in time. That means, the in-degree growth for each group is linear. Let us assume that the in-degree growth rate of group a is denoted by C_a:

$$K_a^{in}(t) = C_a K^{in}(t) \tag{A7}$$

Since the in-degree growth remains constant over time, we can calculate C_a in the empirical network by summing all in-degrees of the group

$$C_a(empirical) = \frac{K_a^{in}}{K^{in}} \tag{A8}$$

Equation (A4) can be rewritten as

$$p_{aa} = f_a \frac{h_{aa} C_a}{h_{aa} C_a + h_{ab}(1 - C_a)} \tag{A9}$$

In empirical networks, p_{aa} represents the probability of a directed edge from class a to class a. This probability is proportional to the number of edges from a to a, normalized by the total number of directed edges that start from a:

$$p_{aa} = \frac{e_{aa}}{e_{aa} + e_{ab}} \tag{A10}$$

We can then calculate Equation (A10) in the empirical network. Finally we use maximum-likelihood estimate to find the best values for h_{aa} and h_{bb} in Equation (A9).

Note that the in-degree growth rate C has an sub-linear relationship to the exponent of the in-degree distribution σ and the exponent of the in-degree growth θ [1]. Thus, another method to retrieve empirical homophily is to first estimate the exponents of the in-degree distributions for minority and majority groups (σ_a and σ_b) and plug that into the equation.

$$p_{aa} = \frac{f_a^2 h_{aa}(1 - \theta_b)}{f_a h_{aa}(1 - \theta_b) + f_b h_{ab}(1 - \theta_a)} \tag{A11}$$

$$p_{bb} = \frac{f_b^2 h_{bb}(1 - \theta_a)}{f_b h_{bb}(1 - \theta_a) + f_a h_{ba}(1 - \theta_b)} \tag{A12}$$

where $\sigma_a = -(\frac{1}{\theta_a} + 1)$ and $\sigma_b = -(\frac{1}{\theta_b} + 1)$.

3. Regression model

We build a `RandomForestRegressor` [2, 3] model to explain rank inequality and inequity given the structure of networks. Features (or independent variables) are transformed by scaling them between zero and one. During training, the model uses $n\_estimators = 100$ and all default values from the `Python` package. We use R^2 scores to evaluate the performance of the 10-fold cross-validated model on the test set. As shown in Table S1, the global model takes into account the overall behavior or trend regardless of top-k ranks, while the local model includes the top-k ranks. We add ϵ as a dummy variable, with randomly generated values between 0 and 1, to compare the importance of each feature to random chance.

We report the importance of features given by the `feature_importances_` property of the `RandomForestRegressor` model. The higher the value, the more important the feature. The importance of a feature is computed as the (normalized) total reduction of the criterion brought by that feature. It is also known as the Gini importance [3].

4. Who-To-Follow produces higher inequality compared to PageRank

In the main manuscript we see that Who-To-Follow (WTF) produces skewer rank distributions compared to PageRank. To understand this behavior, we need to first understand how the algorithms work. PageRank scores reflect the *global* importance of nodes in the network, and this global importance is mostly determined by in-degree [4] and the age of nodes [5]. On the other hand, the WTF score of a node is the number of times the node appears in the top-10 recommendation across all nodes in the network. This top-10 is determined by the circle-of-trust of each node, similar to a Personalized PageRank. This means that this top-10 contains the most visited nodes by a random walker that always starts at the node who is getting the recommendation. Thus, that (local) top-10 will be highly influenced by in-degree too. However, since the WTF score counts the number of times a node appears as a recommendation, it is likely that the highest WTF scores refer to high degree nodes due to preferential attachment. Therefore, the high inequality produced by WTF can be explained by the fact that WTF combines a local random walk with a global count.

Appendix B: Additional Tables and Figures

TABLE S1: **Regression models.** Dependent and independent variables of the four models of interest: Global/Local inequality (Gini) and inequity (ME). We add the dummy variable ϵ (with randomly generated values between 0 and 1) to verify whether the network-based features are better than random or not.

Type	Dependent variable (Y)	Independent variable (Xs)
Global	$Gini$<br>ME	f_m, h_{MM}, h_{mm}, ϵ
Local	$Gini_k$<br>ME_k	f_m, h_{MM}, h_{mm}, k, ϵ

TABLE S2: **10-fold cross-validation for WTF.** We use a Random Forest Regressor to assess feature importance and report the mean and standard deviation of the out-of-sample R^2. Features are ranked in descending order based on their mean importance (from left to right) and highlighted if their importance represents at least 50% of the total importance. Features with a mark (*) are less important than random ϵ. Corr shows the disparity as the Spearman correlation between inequality and inequity scores (p-values ≈ 0).

Type	Outcome	Corr.	R^2	Feature	Importance
**Global**	$Gini$	0.29	0.35 (0.03)	ϵ, h^*_{MM}, h^*_{mm}, f^*_m	0.37, 0.27, 0.22, 0.14
	ME		0.92 (0.01)	$\boldsymbol{h_{MM}}, h_{mm}, f_m, \epsilon$	0.51, 0.37, 0.07, 0.05
**Local**	$Gini_k$	0.06	0.86 (0.00)	$\boldsymbol{k}, \epsilon, h^*_{MM}, h^*_{mm}, f^*_m$	0.86, 0.08, 0.02, 0.02, 0.01
	ME_k		0.85 (0.01)	$\boldsymbol{h_{MM}}, \boldsymbol{h_{mm}}, k, \epsilon, f^*_m$	0.43, 0.31, 0.11, 0.08, 0.07

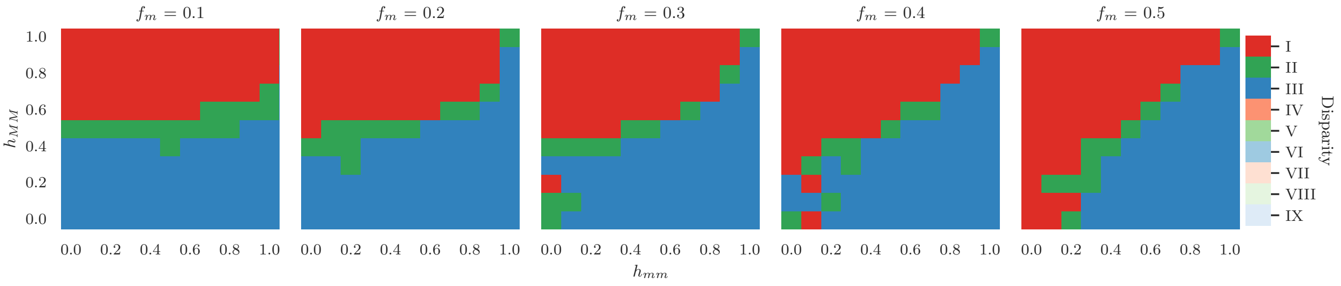

FIG. S1: **The effects of homophily and fraction of minorities in the global disparity of WTF.** Columns represent the fraction of minorities in the network, x-axis indicates the homophily within minorities, and y-axis the homophily within majorities. Colors denote the region where the disparity lies according to our interpretation (see Figure 2 in main article). As in the case of PageRank (cf. Figure 4 in main article), we see that, on average, there is never low global inequality. Also, depending on the level of homophily within groups, minorities on average can be under-represented (region I, red), or over-represented (region III, blue). Note that the fair case (region II, green) rarely occurs.

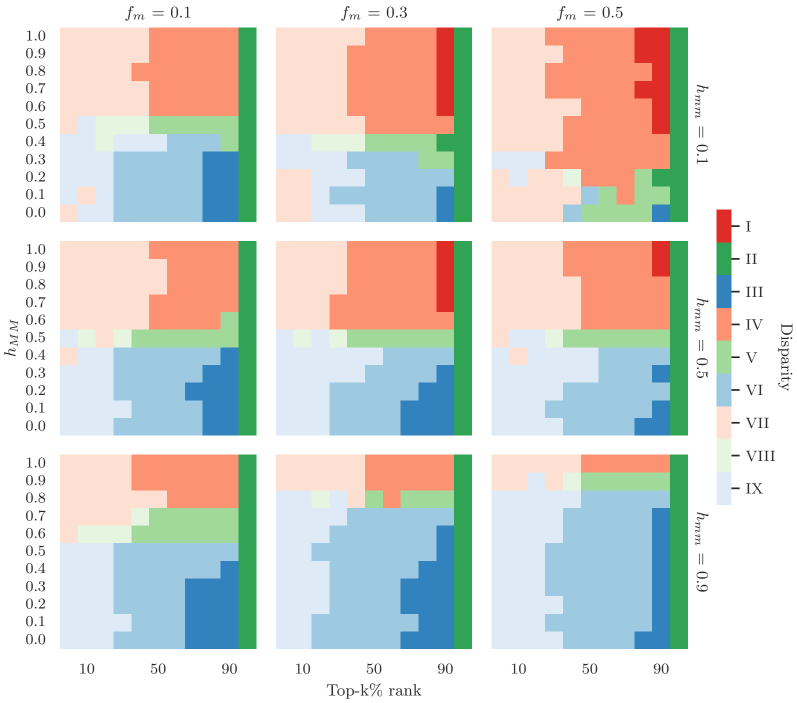

FIG. S2: **The effects of homophily and fraction of minorities in the local disparity of WTF.** Columns represent the fraction of minorities (10%, 30% and 50%) and rows show homophily within minorities (from top to bottom: heterophilic, neutral and homophilic). The x-axis denotes the top-k% rank and the y-axis shows homophily within majorities. Colors refer to the regions of disparity (see Figure 2 in main article). As in the case of PageRank (cf. Figure 5 in main article), we see that the minority suffers most (red) when the majority is homophilic and the minority is either heterophilic or neutral. Also, inequity remains mostly consistent regardless of top-k%. In contrast to PageRank (up to top-5%), WTF manages to capture nodes with very similar ranking scores (roughly) up to the top-30% (i.e., Gini is low, regions VII, VIII, IX).

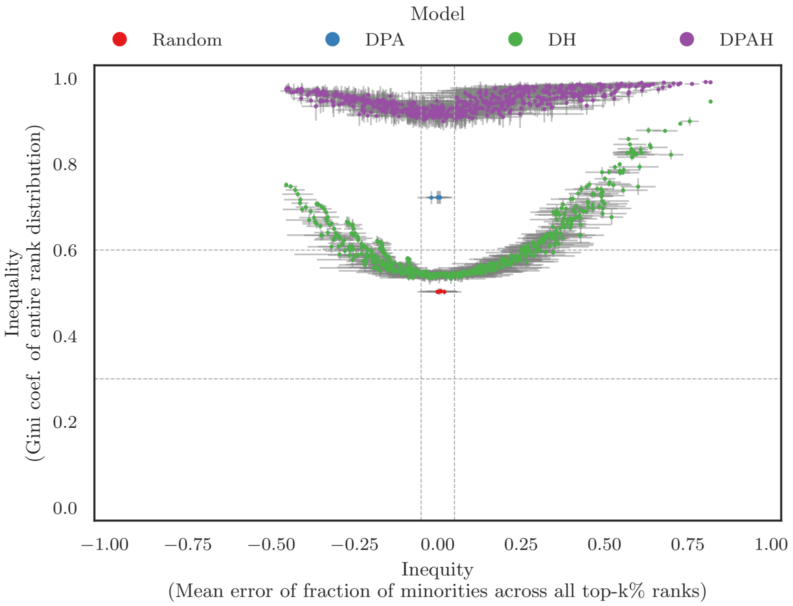

FIG. S3: **The effects of homophily and preferential attachment in the global disparity of WTF.** We generated directed networks using four different models of edge formation. DPA: only preferential attachment. DH: only homophily. DPAH: our proposed model that combines DPA and DH. Random: a baseline where nodes are connected randomly. Compared to PageRank (cf. Figure 6 in main article), all models generate higher inequality (y-axis), whereas inequity remains similar. Vertical and horizontal error bars represent the standard deviation over 10 runs of the Gini and ME, respectively.

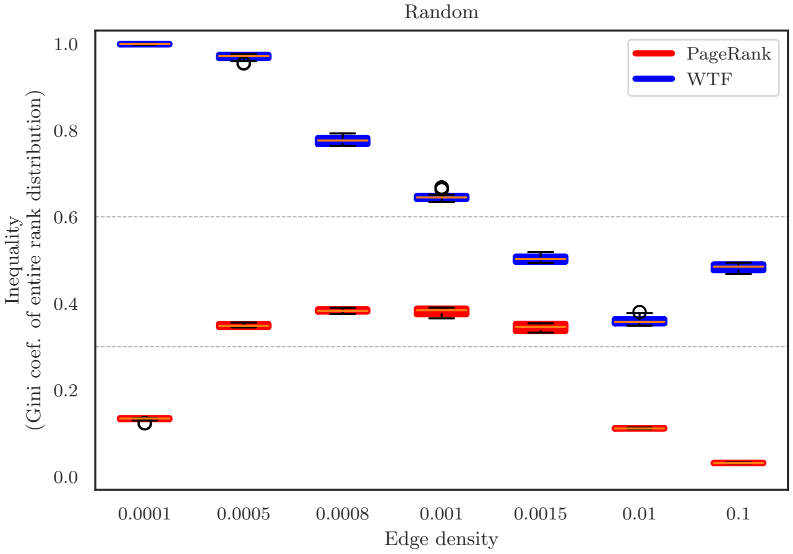

FIG. S4: **Global inequality on Random networks as a function of edge density.** We generate directed Erdős-Rényi networks to demonstrate how the global inequality (y-axis) varies with respect to the edge density (x-axis) of the network. For each density value we generate networks with different fractions of minorities and 10 epochs. Note that $d = 0.0015$ corresponds to the Random networks used in the main experiments. Inequality computed on the PageRank distribution is shown in red, while the inequality on WTF is shown in blue. We see different trends for each algorithm. First, the inequality (Gini coefficient) of PageRank is very low when the edge density is extreme (i.e., either too low or too high). This means that in these regimes most nodes are similarly important regardless of the magnitude of their degrees. Second, the inequality of WTF is in general negatively correlated with density (i.e., the lower the density, the higher the inequality [6]). However, in the extreme case of denser networks (i.e., $d = 0.1$), inequality raises. Recall that ranking *inequity* is very close to zero ($ME \approx 0$) in random networks. Further studies are required to analytically understand the limits of inequality with respect to density.

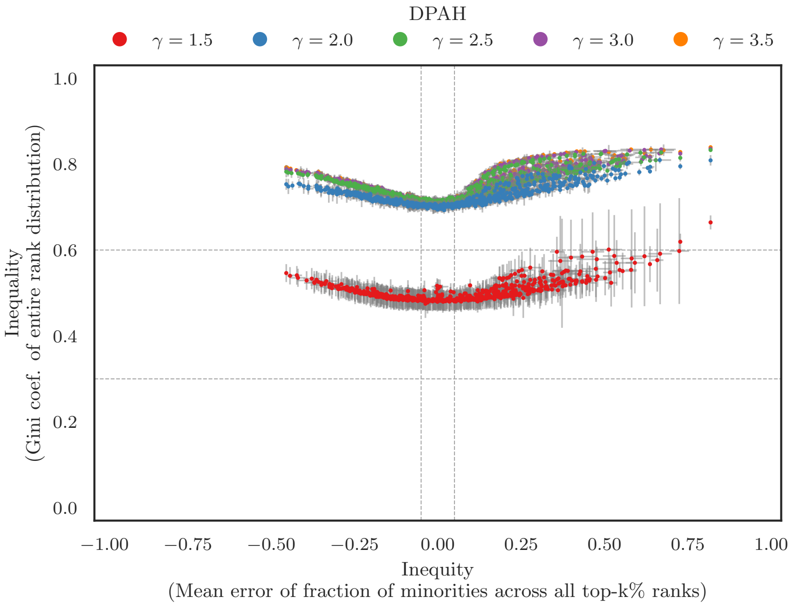

FIG. S5: **The effects of symmetric node activity in the global disparity of PageRank.** We generate DPAH networks by varying f_m, h_{MM}, h_{mm}, and fixing number of nodes $n = 2000$ and edge density $d = 0.0015$. Each color represents the activity of nodes as the out-degree exponents of the networks $\gamma = \gamma_M = \gamma_m \in \{1.5, 2.0, 2.5, 3.0, 3.5\}$. We see that by reducing the out-degree exponent in the DPAH networks (from $\gamma = 3.5$ to $\gamma = 1.5$), we reduce inequality (vertical axis), and inequity remains stable. Vertical and horizontal error bars represent the standard deviation over 10 runs of the Gini and ME, respectively.

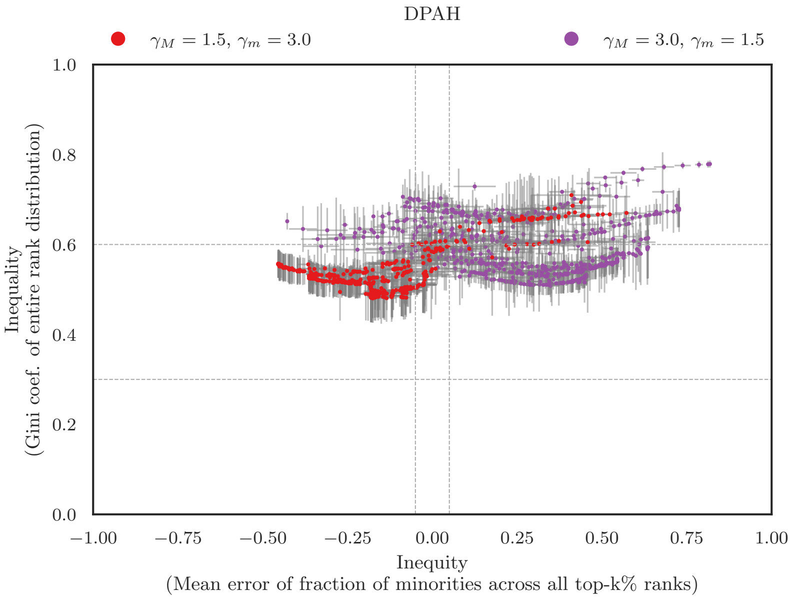

FIG. S6: **The effects of asymmetric node activity in the global disparity of PageRank.** Similar to Figure S5, we generate DPAH networks by varying f_m, h_{MM}, h_{mm} and fixing number of nodes $n = 2000$ and edge density $d = 0.0015$. Additionally, we adjust γ_M and γ_m, the activity of majority and minority groups, respectively. Red represents networks where the majority is more active (higher out-degree) than the minority. In contrast, purple represents networks where the minority is more active than the majority. In comparison with their counterpart $\gamma = \gamma_M = \gamma_m = 3.0$ in Figure S5, we see that a more active minority ($\gamma_M = 3.0$, $\gamma_m = 1.5$), reduces inequality (lower Gini) by amplifying its visibility in the rank (positive ME). Conversely, a more active majority ($\gamma_M = 1.5$, $\gamma_m = 3.0$) can amplify minority representation at the cost of increasing inequality. However, in general, active majorities benefit themselves in the rank. Vertical and horizontal error bars represent the standard deviation over 10 runs of the Gini and ME, respectively.

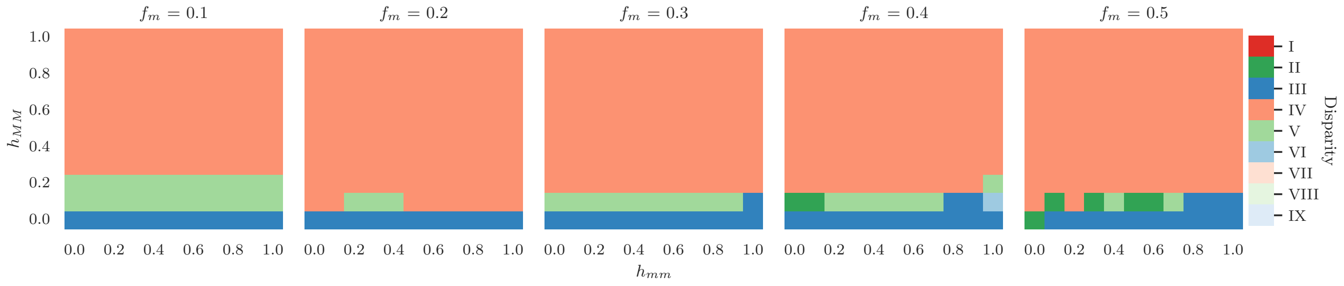

FIG. S7: **Disparities caused by an active majority group on PageRank as a function of homophily and group size.** Columns represent the fraction of minorities in the network, x-axis indicates the homophily within minorities, and y-axis the homophily within majorities. Colors denote the regions where the disparity lies according to our interpretation (see Figure 2 in main article). These results correspond to the red data points in Figure S6, $\gamma_M = 1.5 < \gamma_m = 3.0$. We see that when the majority is more active than the minority, the algorithm reduces the representation of minorities in almost all combinations of homophily and group size. The exception lies in extreme heterophilic majorities. In this case, the ranking may amplify or replicate the visibility of minorities in the rank.

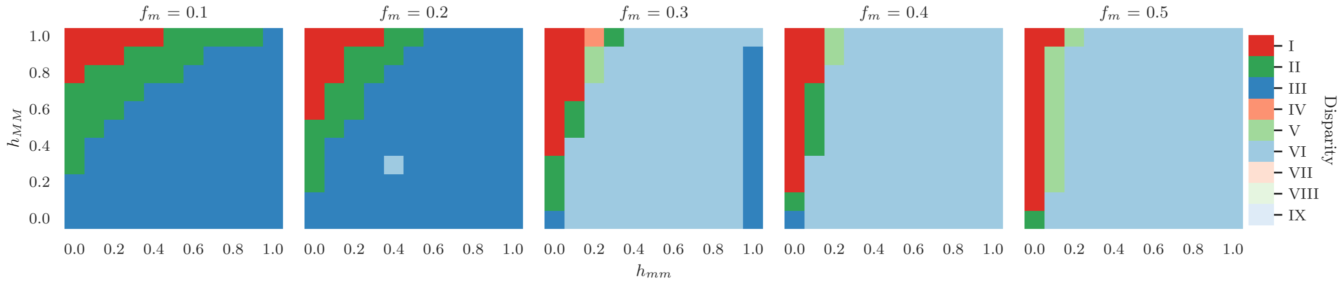

FIG. S8: **Disparities caused by an active minority group on PageRank as a function of homophily and group size.** Columns represent the fraction of minorities in the network, x-axis indicates the homophily within minorities, and y-axis the homophily within majorities. Colors denote the regions where the disparity lies according to our interpretation (see Figure 2 in main article). These results correspond to the purple data points in Figure S6, $\gamma_M = 3.0 > \gamma_m = 1.5$. We see that when the minority is more active than the majority, the algorithm amplifies the representation of minorities in the rank in almost all combinations of homophily and group size. The exception is an interplay between the fraction of minorities and the homophily of the majority group when minorities are heterophilic.

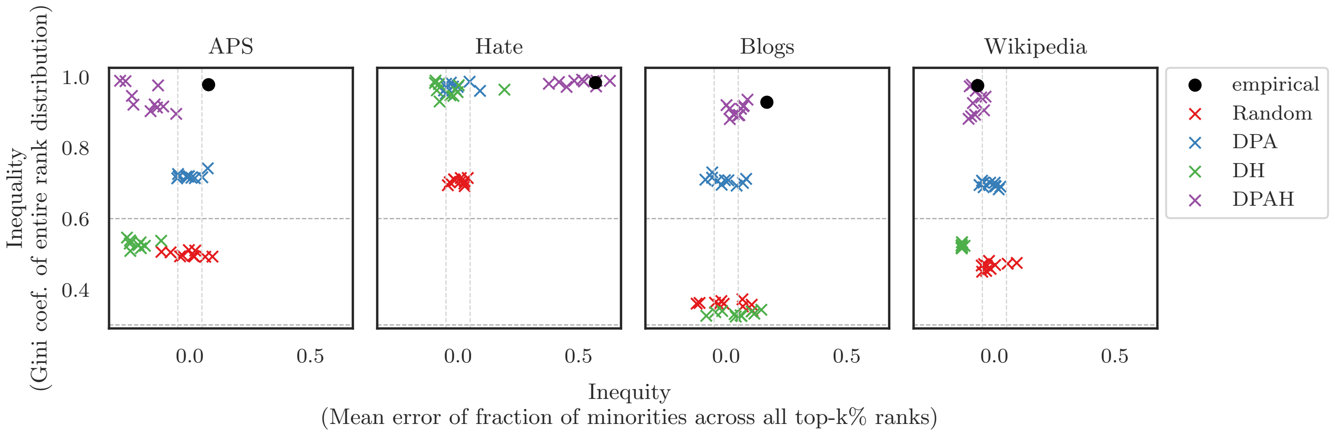

FIG. S9: **Global disparity in WTF on empirical networks.** Each column represents an empirical network. Citation/retweet networks (APS and Hate) and Hyper-link networks (Blogs and Wikipedia). Inequality and inequity are shown in the y- and x-axis, respectively. The disparities in ranking that we see in these empirical networks are best explained by preferential attachment and homophily DPAH.

[1] F. Karimi, M. Génois, C. Wagner, P. Singer, and M. Strohmaier, Scientific reports **8** (2018).
[2] F. Pedregosa, G. Varoquaux, A. Gramfort, V. Michel, B. Thirion, O. Grisel, M. Blondel, P. Prettenhofer, R. Weiss, V. Dubourg, et al., Journal of Machine Learning Research **12**, 2825 (2011).
[3] Scikit-learn, *sklearn.ensemble.randomforestregressor*, `https://scikit-learn.org/stable/modules/generated/sklearn.ensemble.RandomForestRegressor.html`, accessed: 2020-11-09.
[4] S. Fortunato, M. Boguñá, A. Flammini, and F. Menczer, in *International workshop on algorithms and models for the web-graph* (Springer, 2006), pp. 59–71.
[5] M. S. Mariani, M. Medo, and Y.-C. Zhang, Scientific reports **5**, 1 (2015).
[6] S. Goswami, C. Murthy, and A. K. Das, Information Sciences **462**, 16 (2018).
